# Incorporation of silica nanoparticles to PLGA electrospun fibers for osteogenic differentiation of human osteoblast-like cells

**DOI:** 10.1093/rb/rby014

**Published:** 2018-06-09

**Authors:** Xing Yang, Yuanyuan Li, Xujie Liu, Qianli Huang, Ranran Zhang, Qingling Feng

**Affiliations:** 1 State Key Laboratory of New Ceramics and Fine Processing, School of Materials Science and Engineering, Tsinghua University, Beijing, China; 2 Department of Stomatology, Shengli Oilfield Central Hospital, Dongying, China; 3 Graduate School at Shenzhen, Tsinghua University, Shenzhen, China; 4 State Key Laboratory of Powder Metallurgy, Central South University, Changsha, China; 5 Key Laboratory of Advanced Materials of Ministry of Education of China, School of Materials Science and Engineering, Tsinghua University, Beijing, China

**Keywords:** composite fibers, silica nanoparticles, osteogenic differentiation, bone tissue engineering

## Abstract

The development of bone tissue engineering scaffolds still remains a challenging field, although various biomaterials have been developed for this purpose. Electrospinning is a promising approach to fabricate nanofibers with an interconnected porous structure, which can support cell adhesion, guide cell proliferation and regulate cell differentiation. The aim of this study is to fabricate composite fibers composed of poly(lactic-co-glycolic acid) (PLGA) and silica nanoparticles (NPs) via electrospinning and investigate the effect of PLGA/SiO_2_ composite fibers on the cellular response of osteoblast-like cells (SaOS-2 cells). SEM and EDX analysis showed that silica NPs were homogenously dispersed in the composite fibers. The mechanical behavior of the fibers showed that silica NPs acted as reinforcements at concentrations of 2.5 and 5 mg/ml. The incorporation of silica NPs led to enhancement of cell attachment and spreading on PLGA/SiO_2_ composite fibers. SaOS-2 cells cultured on PLGA/SiO_2_ composite fibers exhibited increased alkaline phosphatase activity, collagen secretion and bone nodules formation. The bone nodules formation of SaOS-2 cells increased along with the amount of incorporated silica NPs. The present findings indicate that PLGA/SiO_2_ composite fibers can stimulate osteogenic differentiation of SaOS-2 cells and may be a promising candidate scaffold for bone tissue engineering.

## Introduction

Bone defects, caused by genetic malformations, trauma, infections or tumors, are very common within the world’s population and are threatening human health [1]. There are several limitations for the treatments of autogenous or xenogeneic bone grafts [2]. Therefore, many kinds of scaffolds have been manufactured with tissue engineering strategies for treating bone defects, such as polymers [3], metals [4] and bio-ceramics [5]. Apart from hydroxyapatite, silica has emerged as a beneficial inorganic component for scaffolds in bone tissue engineering. As a kind of bio-ceramics, silica has been reported to have stimulatory effect on the osteogenic differentiation of bone-forming cells [6–8]. For instance, our recent study showed that the silica nanoparticles (NPs) significantly increased the alkaline phosphatase (ALP) activity and enhanced mineralization of the human mesenchymal stem cells (hMSCs) [9]. Similarly, Ha *et al.* found that silica NPs could stimulate osteogenic differentiation of mouse bone marrow stromal cells and MC3T3-E1 pre-osteoblast cells through enhancing ALP activity, bone nodules formation and bone-related genes expression [10].

It is known that natural bone matrix is a polymeric/inorganic composite material made of collagen and apatite [11]. Composite materials consisting of biodegradable bioceramic NPs and polymers appear to be a better choice as scaffolds for bone tissue repair and regeneration [12]. Poly(lactic-co-glycolic acid) (PLGA) is one of the most popular and widely used synthetic polymers for bone tissue engineering, since it is a well-known biocompatible and biodegradable polymer approved by US Food and Drug Administration (FDA) and its degradation rate can be controlled by the ratio of lactic acid and glycolic acid [13, 14]. PLGA scaffolds have been proved to beneficial to cell adhesion and proliferation of bone-related cells [13, 15, 16]. Taken together, due to the attractive bioactivities, composite scaffold composed of silica NPs in PLGA matrix may be a candidate for bone tissue engineering.

The ultimate goal of the development of scaffolds for bone tissue engineering is mimicking the structural and physicochemical features of extracellular matrix (ECM), since ECM is the natural scaffold for most tissues [13]. ECM consists of a network of nanosized proteins and glycosaminoglycans with a highly porous structure and wide distribution of the pore size [11, 17]. Hence, the electrospinning technique is introduced to fabricate polymeric scaffolds for tissue engineering. Electrospinning can easily manufacture polymeric scaffolds with a nanofibrous structure and large size range (several nanometers to hundreds of microns) via altering the composition of polymer solution and processing parameters [11]. Several electrospun composite scaffolds such as PLGA/HA, PCL/HA and PLLA/collagen have been developed for bone tissue engineering [16, 18, 19]. However, the effect of electrospun composite scaffold composed of PLGA and silica NPs on osteogenesis has not been investigated. It is therefore of great interest to fabricate a composite scaffold containing PLGA and silica NPs using electrospinning technique for control of osteogenesis.

In present study, composite fibers consisting of PLGA and silica NPs were fabricated through electrospinning process. We investigated the effect of PLGA/SiO_2_ composite fibers on adhesion, proliferation and osteogenic differentiation of osteoblast-like cells.

## Materials and methods

### Preparation and characterization of silica NPs

The silica NPs (∼50 nm) were synthesized according to the modified Stöber method as our previous work [9, 20]. In brief, 10.6 mg of fluorescein isothiocyanate and 24 μl of (3-aminopropyl)triethoxysilane in 2 ml of absolute ethanol was stirred for 16 h to get Solution A. Subsequently, 1.65 ml of tetraethyl orthosilicate and 0.5 ml of Solution A were added to premixed ethanol (39.625 ml) and ammonia (2.125 ml) with magnetic stirring for 24 h. Finally, 0.5 ml of tetraethyl orthosilicate was added and magnetic stirring was continued for another 24 h. The silica NPs were then centrifuged, washed with deionized water and dried at room temperature overnight. Field emission scanning electron microscopy (FESEM; Merlin Compact, Zeiss, German) was used to observe the size and morphology of the NPs. The size distribution of the NPs was measured using Nano measurer 1.2 software.

### Preparation and characterization of fibers

PLGA and PLGA/SiO_2_ composite fibers (PLGA/SiO_2_-2.5, PLGA/SiO_2_-5) were fabricated by electrospinning process. PLGA pellets (PLA/PGA = 75:25, Daigang, China) were dissolved in 1,1,1,3,3,3-hexafluoro-2-propanol at a *w*/*v* ratio of 20%. Silica NPs at various concentrations (2.5 and 5 mg/ml) were then added and the mixture was continuously stirred for 1 h. For electrospinning process, the polymer solution was loaded in a 5-ml syringe capped with a 27-G needle. A distance of 12 cm was maintained between the needle and the collector. The polymer solution was sprayed onto aluminum foil covering a rectangular collector at 18.0 kV with a flow rate of 0.6 ml/h. The electrospun fibers were subsequently dried at room temperature for additional analysis. For several testing methods during cell culture, the composite fibers were prepared on pure Ti discs (10 mm in diameter, 2 mm thick). The morphology of fibers was observed using FESEM (Merlin Compact) with EDX analysis. Each specimen was coated with a thin layer of Pt using an ion-sputtering coater. The mechanical properties of PLGA and PLGA/SiO_2_ composite fibers were measured via nanoindentation. The nanoindentation experiments were carried out using a Keysight G200 Nanoindenter (USA) with a three-sided pyramidal Berkovich tip at room temperature.

### Si ions release

In order to investigate the *in vitro* degradation behavior, PLGA/SiO_2_-2.5 and PLGA/SiO_2_-5 composite fibers were immersed in 0.5 ml McCoy’s medium (Gibco) at 37°C on a shaker for 7 days. The supernatant was collected and the concentration of free silicon was measured by inductively coupled plasma atomic emission spectrometer (ICP-AES).

### Cell culture

SaOS-2 cells, purchased from China Infrastructure of Cell Line Sources, were employed as osteoblast model in this study because of their similarities with human osteoblasts regarding the ALP activity, gene regulation and mineralization potential [21]. The cells were maintained in normal culture medium composed of 15% fetal bovine serum (FBS), 1% penicillin–streptomycin and 84% McCoy’s medium (Gibco) under standard culture condition (5% CO_2_, 37°C and 100% humidity). The culture medium was refreshed every 2 days.

### Cell attachment

To investigate cell attachment, SaOS-2 cells were seeded onto PLGA, PLGA/SiO_2_-2.5 and PLGA/SiO_2_-5 composite fibers in 48-well plates at a density of 1.5 × 10^4^ cells/cm^2^. After 8 and 24 h, the cells were fixed with 4% paraformaldehyde for 30 min, permeabilized with 0.1% Triton X-100 (Sigma, USA) for 5 min and blocked with PBS containing 1% bovine serum albumin. Afterwards, the cells were stained with 5 μg/ml phalloidine-TRITC (Sigma) for 40 min and 4 μg/ml 4′,6-diamidino-2-phenylindole (DAPI) for 10 min at room temperature. The stained samples were visualized and photographed by a confocal-laser scanning microscopy (CLSM, CarlZeiss710, Germany).

### Cell viability

Cell viability on PLGA, PLGA/SiO_2_-2.5 and PLGA/SiO_2_-5 composite fibers was determined by Cell Count Kit-8 (CCK-8, Dojindo, Japan) assay as described previously [22]. On Day 1, 3 and 5, CCK-8 solution (10% CCK-8 and 90% normal culture medium) was added into each well and incubated at 37°C for 2 h. The absorbance (OD) of the solution at 450 nm was then measured using a microplate reader (PerkinElmer, USA).

### Cell differentiation

On Day 5, cells cultured on different fibers (PLGA, PLGA/SiO_2_-2.5 and PLGA/SiO_2_-5) were washed three times with PBS and then lysed with RIPA lysis buffer (Beyotime, China). According to the manufacturer’s protocol, ALP activity in the lysate was assessed using an ALP testing kit (Jiancheng, China). Total protein content was measured with the BCA assay kit (Aidlab, China) and the ALP activity was normalized to the total protein content.

Collagen secretion on different fibers was determined by Sirius Red staining after 10 days of culture. In brief, the cells were washed three times with FBS, fixed with 4% paraformaldehyde and stained with Sirius Red. For quantitative analysis, the stain on specimens was eluted with 50% 0.2 M NaOH in methanol for 10 min and the absorbance (OD) at 570 nm was measured using a microplate reader (PerkinElmer).

Alizarin Red S staining was performed to evaluate the ECM mineralization by the cells on different fibers. The cells were cultured in osteogenic medium (normal culture medium containing 10^−7^ ^ ^M dexamethasone, 50 μg/ml ascorbic acid and 10 mM β-glycerophosphate disodium). On Day 10 after osteogenic induction, the cells were fixed using 4% paraformaldehyde for 30 min and stained with 1% Alizarin Red S at pH 4.2 for 15 min. The stained samples were photographed with a digital camera. For quantitative analysis, the stain on specimens was dissolved in 10% (*w*/*v*) cetylpyridinium chloride monohydrate in 10 mM sodium phosphate buffer (pH 7) for 15 min at 37°C and the absorbance (OD) was measured using a microplate reader (PerkinElmer) at 570 nm.

### Statistical analysis

All results were represented as the mean ± standard deviation (SD) and statistical analysis was conducted using SPSS (Ver. 15.0.1) software. One-way analysis of variance (ANOVA) followed by the least significant difference method was used to evaluate differences between groups. The *P* < 0.05 was considered statistically significant.

## Results and discussion

### Characterization of silica NPs and fibers

**Figure 1.**
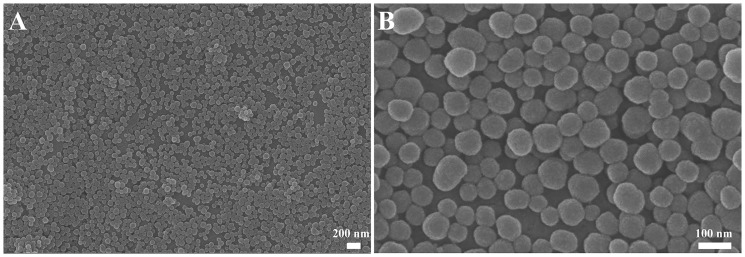
SEM Images of spherical silica NPs. The average size of silica NPs is ∼50 nm

The morphology of prepared silica NPs observed using SEM is shown in Fig. 1. The synthesized silica NPs are spherical in shape with homogenous size distribution. The average diameter of the silica NPs is 53.2 ± 4.3 nm, which is determined by analysis of 200 NPs of SEM images. Because of their spherical shape, the size of silica NPs refers to the diameter of these particles.

**Figure 2.**
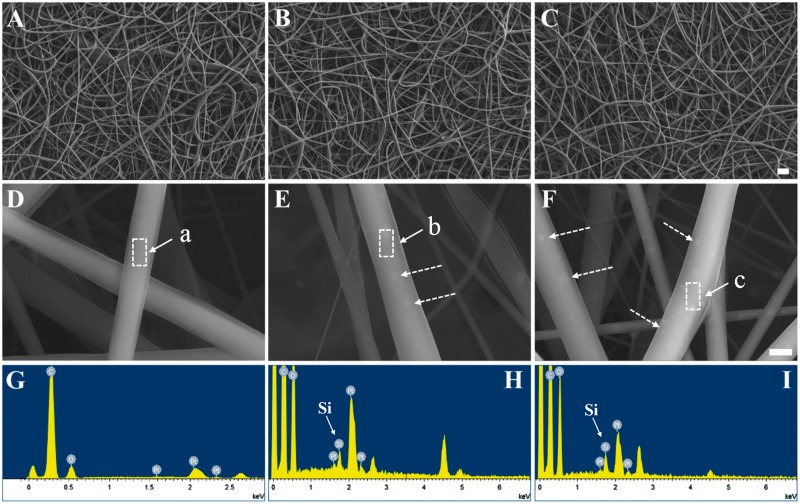
SEM images of PLGA (**A**, **D**), PLGA/SiO_2_-2.5 (**B**, **E**) and PLGA/SiO_2_-5 (**C**, **F**) fibers and EDX analysis of area a (**G**), b (**H**) and c (**I**). The composite fibers show silica NPs on the surface (dotted arrows in E and F). The bar for A, B and C is 5 μm and for D, E and F is 500 nm

Surface morphology of the fibers (PLGA, PLGA/SiO_2_-2.5 and PLGA/SiO_2_-5) is confirmed by SEM (Fig. 2). As shown in Fig. 2A–C, the fibers in three groups have random orientation with uniform fibrous feature on the surface and interconnected porous structure. The diameter of PLGA and PLGA/SiO_2_ fibers, which is determined by analysis of the SEM images, is in a range of 500–800 nm. Several process parameters, for instance, polymer solution concentration, voltage, humidity, viscosity and temperature can influence the morphology and diameter of electrospun fibers [23]. Previous studies showed that the fibers with nanometer-scale dimensions, porous structure and highly nanofibrous topographies were able to modulate cellular responses of a wide variety of cell types [17, 24, 25].

**Figure 3.**
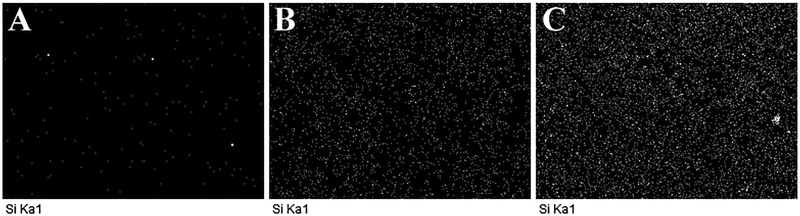
EDX mapping of silicon for PLGA (**A**), PLGA/SiO_2_-2.5 (**B**) and PLGA/SiO_2_-5 (**C**)

The silica NPs were seen on the surface of PLGA/SiO_2_-2.5 and PLGA/SiO_2_-5 composite fibers, indicated by white arrows in Fig. 2E and F. Furthermore, the elemental weight percent and distribution of silicon on the composite fibers were determined by EDX analysis (Figs 2G–I and 3). Table 1 shows that the elemental weight percent of silicon is 0.55% for PLGA/SiO_2_-2.5 and 1.11% for PLGA/SiO_2_-5. As shown in Fig. 3, PLGA/SiO_2_ composite fibers show strong signals of silicon, while, negligible signals of silicon are collected on the surface of PLGA fibers. In addition, the silica NPs seem to be homogenously dispersed on the surface of the composite fibers since EDX mapping shows uniform distribution of silicon (Fig. 3B and C).

**Table 1.** Elemental weight percent of the fibers determined by EDX

Elemental weight percent (%)	C	O	Pt	Si
PLGA	61.99	27.06	10.95	ND
PLGA/SiO_2_-2.5	60.21	27.34	11.90	0.55
PLGA/SiO_2_-5	61.03	27.61	10.25	1.11

The elastic modulus and hardness measured by nanoindentation is shown in Table 2. The addition of silica NPs increased both elastic modulus and hardness of PLGA fibers. PLGA fibers showed an average modulus and hardness of 1.40 ± 0.30 and 0.22 ± 0.03 GPa, respectively. PLGA/SiO_2_-2.5 and PLGA/SiO_2_-5 composite fibers displayed a significant increase of 57 and 202% in modulus compared with that of PLGA fibers. Moreover, the PLGA/SiO_2_-5 group exhibited the highest hardness, followed by PLGA/SiO_2_-2.5 and PLGA in order. The results of elastic modulus and hardness of the PLGA and PLGA/SiO_2_ composite fibers suggested that silica NPs at concentrations of 2.5 and 5 mg/ml had a reinforcing effect on the polymer matrix. Similar effect could be found in previous studies [26–28], where the addition of nano-hydroxyapatite resulted in a significant increase in mechanical properties of PLGA, PCL and collagen.

**Table 2.** Nanoindentation data of the fibers

Mechanical properties	Modulus (GPa)	Hardness (GPa)
PLGA	1.40±0.30	0.22±0.03
PLGA/SiO_2_-2.5	2.20±0.46*	0.31±0.04*
PLGA/SiO_2_-5	4.23±0.35**	0.42±0.04**

* *P* < 0.05 compared with the PLGA group.

** *P* < 0.01 compared with the PLGA group.

### In vitro Si ion release

**Figure 4.**
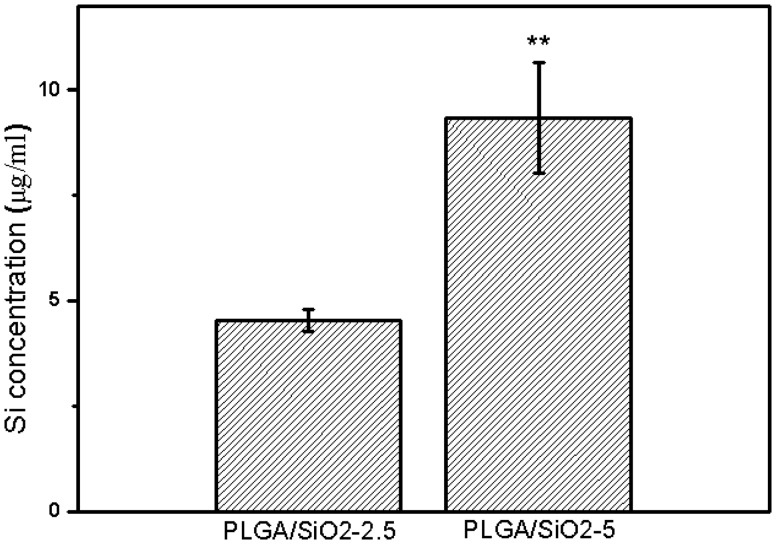
Release of Si ions from PLGA/SiO_2_-2.5 and PLGA/SiO_2_-5 fibers in McCoy’s medium at 37°C for 7 days. Values are expressed as mean ± SD (*n* = 3 for each sample, **P < 0.01)

The degradation of silica NPs on PLGA/SiO_2_ composite fibers was investigated using ICP-AES. As shown in Fig. 4, after 7 days of soaking, the concentration of Si ions (in the form of ${\text{SiO}}_{4}^{4-}$) released from PLGA/SiO_2_-2.5 and PLGA/SiO_2_-5 is 4.5 ± 0.26 and 9.3 ± 1.3 μg/ml, respectively. The PLGA/SiO_2_-5 fibers release approximately two times Si ions as PLGA/SiO_2_-2.5 group, which is consistent with the ratio of the original concentration of silica NPs added to PLGA/SiO_2_-2.5 and PLGA/SiO_2_-5 fibers. The result indicates that silica NPs on both PLGA/SiO_2_-2.5 and PLGA/SiO_2_-5 fibers possess degradability. As a trace element, silicon is beneficial to bone health and has been proven to play a key role in stimulating the osteogenesis [7, 29]. The role of released Si ions in regulating osteogenic differentiation of SaOS-2 cells is discussed in the cell differentiation section.

### Cell attachment and spreading

**Figure 5.**
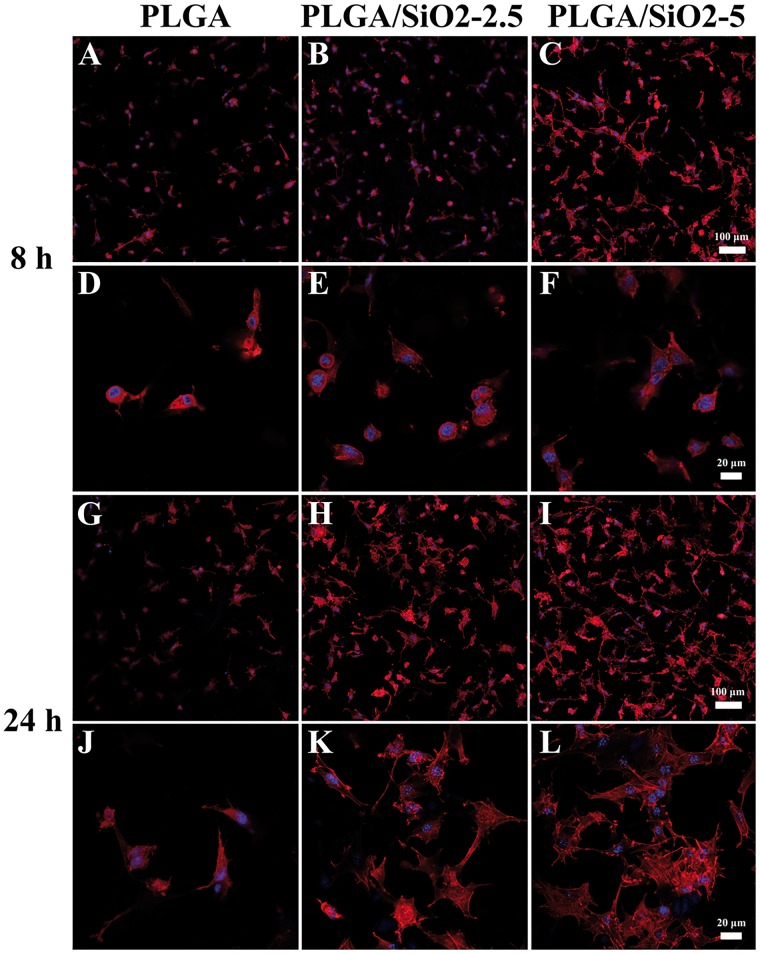
CLSM Images of SaOS-2 cells after 8 and 24 h of culture on PLGA, PLGA/SiO_2_-2.5 and PLGA/SiO_2_-5 fibers. F-actin was stained with phalloidine-TRITC (red) and cell nucleus was stained with DAPI (blue)

Cell attachment and spreading, which can mediate the sequential cellular responses including proliferation and differentiation, are critical for the functional development of anchorage-dependent cells [30, 31]. Figure 5 shows the staining images of cytoskeletons and cell nuclei of SaOS-2 cells on PLGA, PLGA/SiO_2_-2.5 and PLGA/SiO_2_-5 fibers after 8 and 24 h of culture. After seeding for 8 h, SaOS-2 cells on PLGA and PLGA/SiO_2_-2.5 fibers exhibit a relative round morphology (Fig. 5D and E), while, cells on PLGA/SiO_2_-5 fibers spread better and there are some cell membrane extension (cell filopodia) (Fig. 5F). After 24 h, most of the cells on PLGA fibers exhibit a spindle shape without obvious formation of actin stress fibers (Fig. 5J). However, SaOS-2 cells on PLGA/SiO_2_ fibers spread to polygonal morphology and form actin stress fibers (Fig. 5K and L). Furthermore, the amount of cells attached on PLGA/SiO_2_ fibers is higher than that on PLGA fibers (Fig. 5A–C and G–I). Collectively, in comparison with the cells on PLGA fibers, cells on PLGA/SiO_2_ fibers exhibit higher density (Fig. 5A–C and G–I) and larger spreading area (Fig. 5D–F and J–L), especially those on PLGA/SiO_2_-5 fibers. These indicate that PLGA/SiO_2_ composite fibers, especially PLGA/SiO_2_-5, are more beneficial for the attachment and spreading of SaOS-2 cells.

Generally, protein adsorption has been proven to be an important determinant of cell adhesion and spreading [32]. The protein adsorption on materials is strongly affected by their surface topography and surface chemistry [33]. In this study, three different fibers displayed similar surface topography (Fig. 2). Hence, the enhanced cell attachment and spreading on PLGA/SiO_2_ composite fibers were probably caused by the altered surface chemistry after silica NPs incorporation. Si-OH groups on biomaterial surfaces are known to be capable of modulating protein adsorption and cellular response [34, 35]. Shie *et al.* reported that the Si–OH layer on calcium silicate cement surface can promote cell attachment and growth through altering the functional presentation of the major integrin-binding domain of adsorbed proteins [36]. It is known that there are abundant Si–OH groups on the surface of silica NPs prepared via the Stöber method [10, 37]. Thus, the Si–OH groups on silica NPs probably result in that PLGA/SiO_2_ fibers are more favorable for cell adhesion and spreading.

### Cell viability

**Figure 6.**
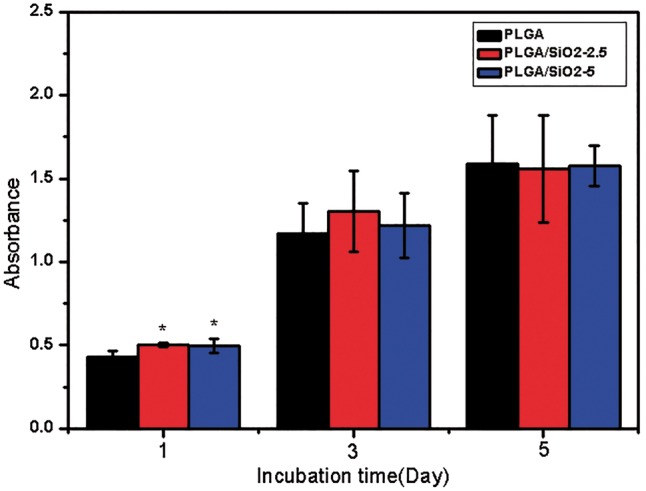
Cell viability detected by CCK-8 assay after 1, 3 and 5 days of culture on PLGA, PLGA/SiO_2_-2.5 and PLGA/SiO_2_-5 nanofibers. Values are expressed as mean ± SD (*n* = 3 for each sample, *P < 0.05)

The cell viability of SaOS-2 cells cultured on PLGA, PLGA/SiO_2_-2.5 and PLGA/SiO_2_-5 fibers after seeding for 1, 3 and 5 days was characterized by CCK8 assay. Figure 6 shows that after 1 day of culture, the cell viability of SaOS-2 cells on PLGA/SiO_2_-2.5 and PLGA/SiO_2_-5 fibers is higher than that on PLGA fibers, which is consistent with the results of cell attachment after 24 h of culture (Fig. 5G–I). As incubation time increases, the cell number increases on all fibers and the viability of SaOS-2 cells shows no significant difference among the three groups at Day 3 and 5. These results indicate that PLGA/SiO_2_ fibers can increase the amount of attached SaOS-2 cells after seeding for 1 day, while, they do not affect the cell viability of SaOS-2 cells after 3 and 5 days of incubation.

### Cell differentiation

Several testing methods, including ALP activity assay, Sirius Red staining and Alizarin Red S staining, were applied to investigate the osteogenic differentiation of SaOS-2 cells on the three fabricated fibers. For Sirius Red staining and Alizarin Red S staining, the PLGA, PLGA/SiO_2_-2.5 and PLGA/SiO_2_-5 fibers were fabricated on pure Ti discs (10 mm in diameter, 2 mm thick). The composite fibers prepared on Ti discs were beneficial to sample handling steps during the staining process.

**Figure 7.**
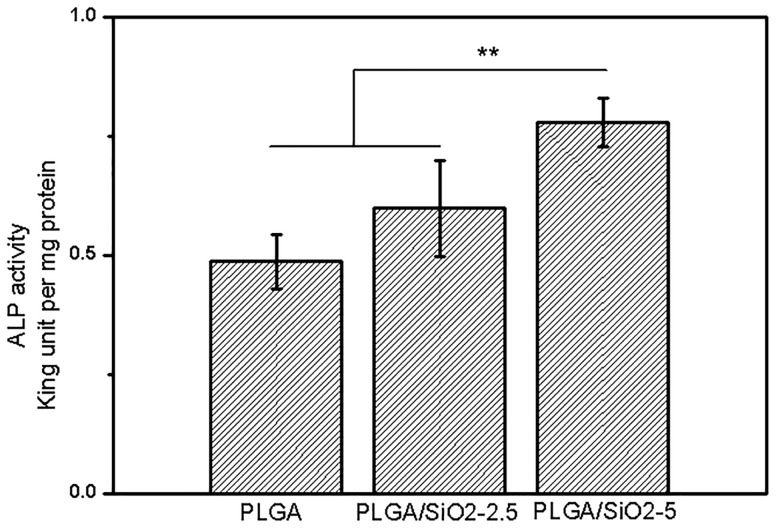
ALP Activity of SaOS-2 cells cultured on PLGA, PLGA/SiO_2_-2.5 and PLGA/SiO_2_-5 fibers for 5 days. Values are expressed as mean ± SD (*n* = 3 for each sample, **P < 0.01)

ALP is an early marker for osteogenic differentiation [9]. The ALP activity of SaOS-2 cells after induction for 5 days on all fibers is presented in Fig. 7. The cells cultured on PLGA/SiO_2_-5 fibers show the highest ALP activity, which increases by roughly 59 and 30% compared with that on PLGA and PLGA/SiO_2_-2.5 fibers, respectively. In addition, the ALP activity of cells on PLGA/SiO_2_-2.5 fibers also shows some extent of increase (∼20%) compared with that on PLGA fibers, although no significant difference is found. The result shows that the addition of SiO_2_ increases the ALP activity of SaOS-2 cells.

**Figure 8.**
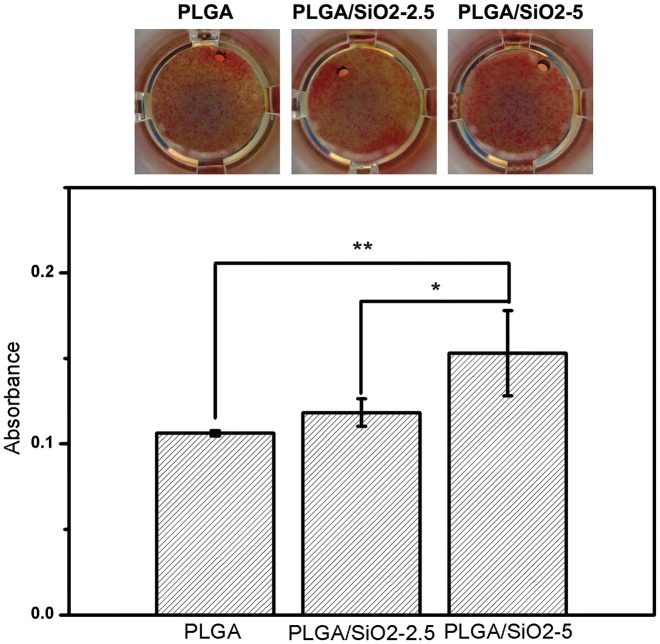
Sirius red staining used for the visualization of collagen deposition and the quantitative result after 10 days of culture on each fibers. Values are expressed as mean ± SD (*n* = 3 for each sample, *P < 0.05 and **P < 0.01)

Sirius Red staining was applied to assess the collagen secretion of SaOS-2 cells after culture for 10 days on various fibers. Sirius Red, an anionic dye, can bind strongly to collagen molecules [38]. The organic bone matrix consists of 85–90% of collagen, therefore the adequate deposition of collagen contributes to bone architecture [38, 39]. As shown in Fig. 8, more intense red color is noticed for cells cultured on PLGA/SiO_2_-5 fibers compared with that on the other two fibers. The quantitative measurement of the Sirius Red staining intensity also shows that the cells on PLGA/SiO_2_-5 fibers secrete more collagen than that on PLGA and PLGA/SiO_2_-2.5 fibers. The incorporation of SiO_2_ to PLGA fibers increases collagen content of SaOS-2 cells in a dose dependent manner as compared with the control group.

**Figure 9.**
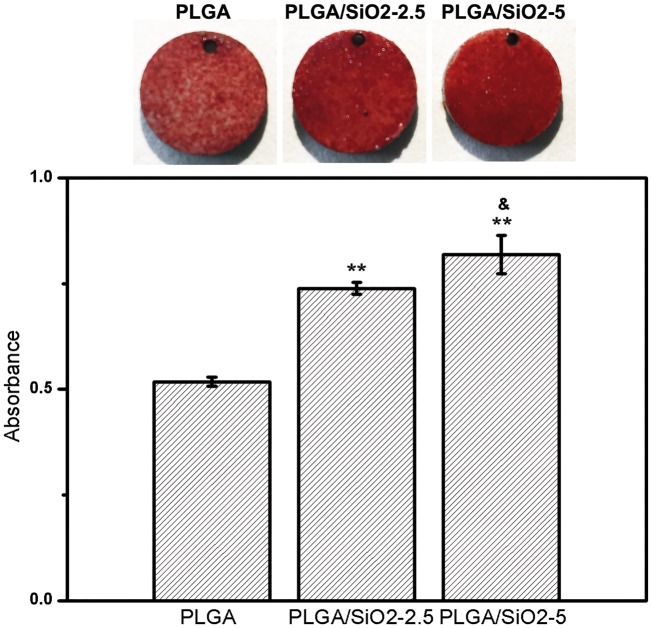
Mineralization nodules stained by alizarin red S staining and the quantitative result after 10 days of osteoinduction on each fibers. Values are expressed as mean ± SD (*n* = 3 for each sample). **P < 0.01 compared with the PLGA group, ^&^*P* < 0.05 compared with the PLGA/SiO_2_-2.5 group

Mineralized nodules formation, a critical indicator at the late stage of osteogenic differentiation [7], was assessed by Alizarin Red S staining on Day 10 after osteogenic induction. As shown in Fig. 9, cells on PLGA/SiO_2_ fibers form larger amount of bone nodules than that on PLGA fibers. The quantitative assay shows a significant increment of 43 and 58% of bone nodules formation by cells on PLGA/SiO_2_-2.5 and PLGA/SiO_2_-5 fibers as compared with that on PLGA fibers. The PLGA/SiO_2_-5 group displays the highest amount of bone nodules formation, followed by PLGA/SiO_2_-2.5 and PLGA in order (PLGA/SiO_2_-5 > PLGA/SiO_2_-2.5 > PLGA). Accordingly, the results suggest that PLGA/SiO_2_-5 fibers are the most effective substrate to stimulate the mineralization of SaOS-2 cells. ALP and collagen have been reported to support the formation of bone nodules during osteogenesis process [40], which partially explains the enhanced mineralization of the cells on PLGA/SiO_2_ fibers.

As mentioned above, these results demonstrate that the fabricated PLGA/SiO_2_ composite fibers can stimulate the osteogenic differentiation of SaOS-2 cells, which is evidenced by enhanced ALP activity, collagen secretion and bone nodules formation. The positive effect of PLGA/SiO_2_ composite fibers on cell differentiation is considered to be related to the enhancement of cell attachment and spreading, and the released Si ions due to the incorporation of silica NPs.

As discussed in section ‘Cell attachment and spreading’, the Si–OH groups on PLGA/SiO_2_ composite fibers are speculated to be the main factor for enhanced cell attachment and accelerated cell spreading. Cell adhesion and spreading are critical for the functional development (including cell proliferation and differentiation) of anchorage-dependent cells. It has been reported that the increased adhesion maturation can promote the osteogenesis of osteoprogenitor cells through enhancing the expression of some osteogenic markers, including runt-related transcription factor 2 (RUNX2) and bone morphogenetic protein 2 (BMP2) [41]. Similarly, Chastain *et al.* reported that cell adhesion to PLGA scaffolds fostered osteogenic differentiation of hMSCs evidenced by higher level of ALP activity and enhanced mineralization [42]. Moreover, alterations in cell spreading can induce modification of intracellular tension and then cause changes in gene expression [33]. The fast cell spreading following cell attachment was reported to enhance osteogenesis via up-regulating the expression of RUNX2 and osteocalcin (OCN) [43]. Thus, the enhanced cell attachment and spreading caused by the incorporation of silica NPs are considered to be responsible for the promotion of osteogenic differentiation of SaOS-2 cells.

The release of Si ions from PLGA/SiO_2_ composite fibers due to dissolution of silica NPs is also presumed to promote the differentiation of SaOS-2 cells. Si ions have been proven to play a key role in stimulating osteogenesis [7]. Our recent study has proved that the Si ions released form silica NPs can be directly linked to the stimulation of osteogenesis [9]. Reffitt *et al.* found that orthosilicic acid (H_4_SiO_4_) could stimulate osteogenic differentiation of several bone-related cell lines via activating the synthesis of collagen I (Col I) and the expression of ALP and OCN [44]. The Si ions released from silica NPs could stimulate the osteogenic differentiation of human bone marrow stromal cells via increasing ALP activity and enhancing the expression of bone-related genes (OCN, RUNX2 and osteopontin (OPN)) [7]. Similarly, the ionic products from Ca_7_Si_2_P_2_O_16_ ceramic were reported to significantly stimulate the proliferation, ALP activity, the expression of osteogenic-related gene (RUNX2, ALP, Col I) and mineralization of periodontal ligament cells (PDLCs) [45]. Recently, several researches have investigated the possible mechanisms involved and found that Si ions can activate osteogenic-related signaling pathways, such as the ERK pathway, TGF-β1 pathway, WNT and SHH pathways [46–48]. In present study, the silica NPs on PLGA/SiO_2_ composite nanofibers can be dissolved and release a certain amount of Si ions (Fig. 4). In this regard, the released Si ions are considered to have stimulatory effect on SaOS-2 cell differentiation.

## Conclusions

In the current work, PLGA/SiO_2_ composite fibers were prepared through electrospinning technique. As shown in SEM and EDX images, silica NPs were homogenously distributed throughout the composite fibers. The incorporation of silica NPs increased the elastic modulus and hardness of the composite fibers and enhanced cell attachment and spreading of SaOS-2 cells on PLGA/SiO_2_ composite fibers. SaOS-2 cells incubated on PLGA/SiO_2_ composite fibers displayed increased ALP activity and collagen secretion. Moreover, bone nodules formation of SaOS-2 cells significantly increased along with the amount of silica NPs in composite fibers. The released Si ions from PLGA/SiO_2_ composite fibers and the enhanced cell attachment and spreading of SaOS-2 cells are considered to be main contributors for the stimulation of SaOS-2 cell differentiation. Our results suggest that incorporating silica NPs into PLGA fibers can promote the osteogenic differentiation of osteoblast-like cells without compromising cell proliferation and such composite fibers may be a good substrate for applications in bone tissue engineering.
